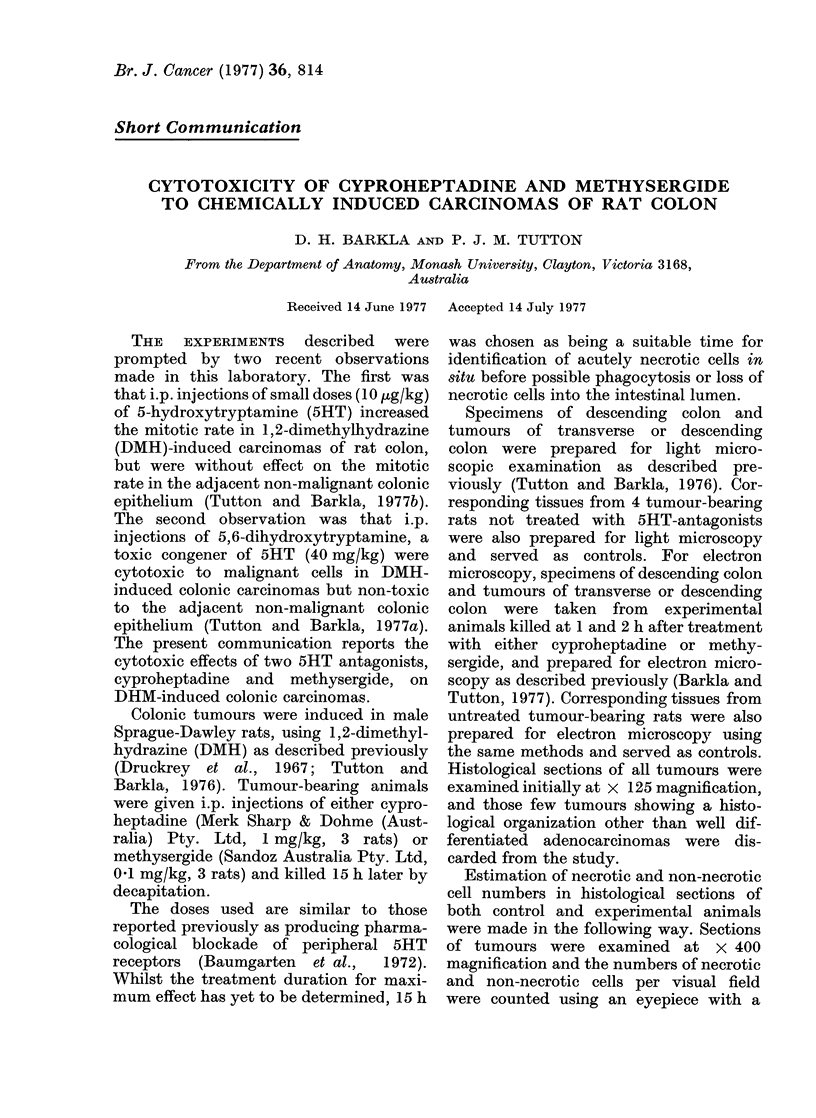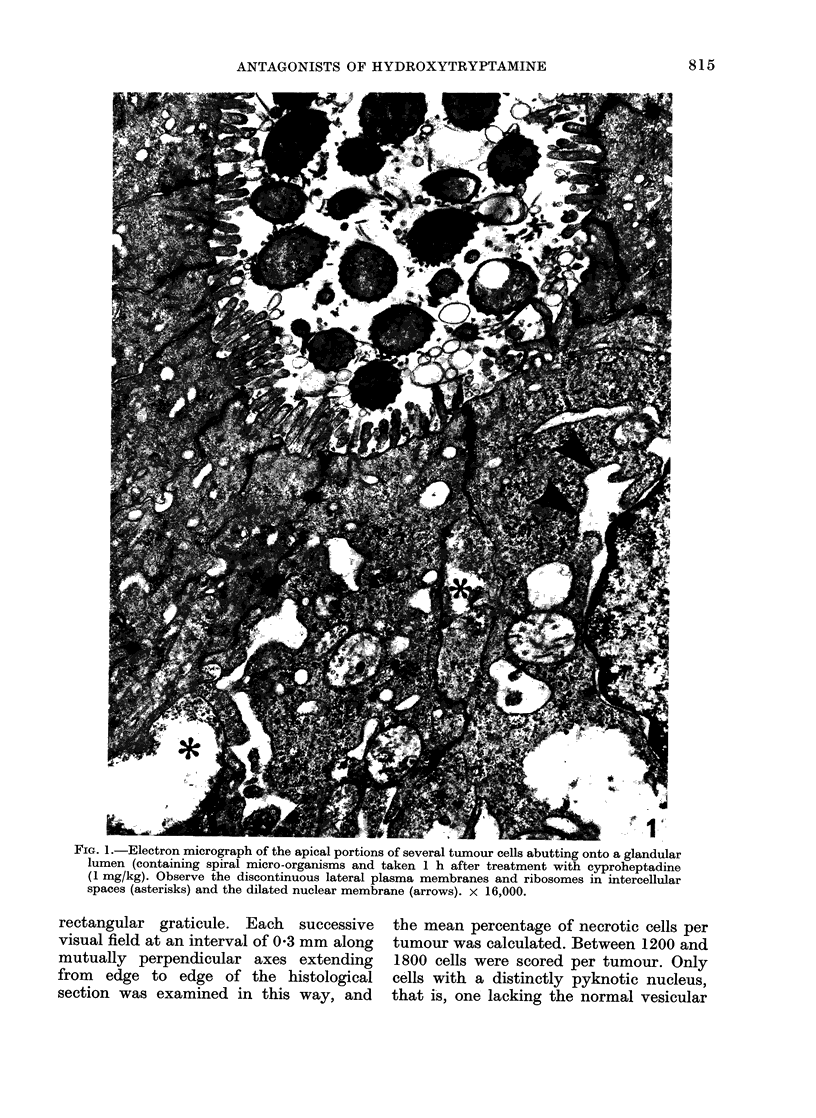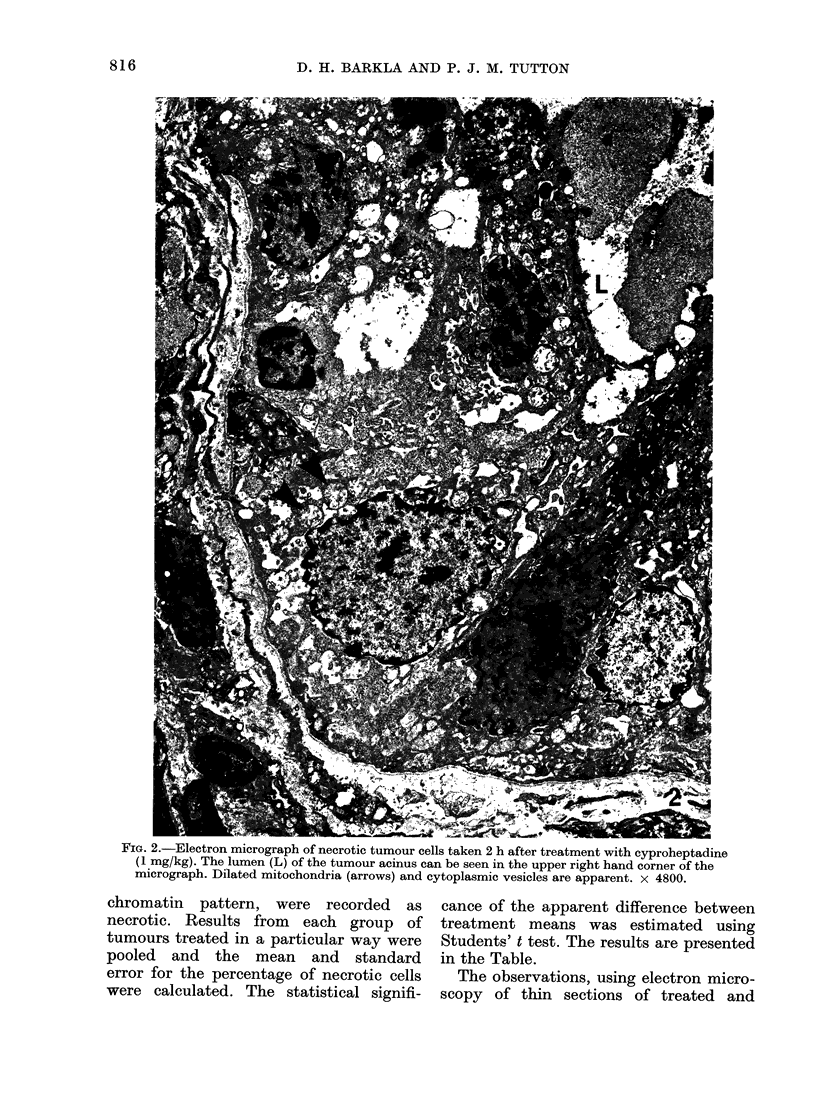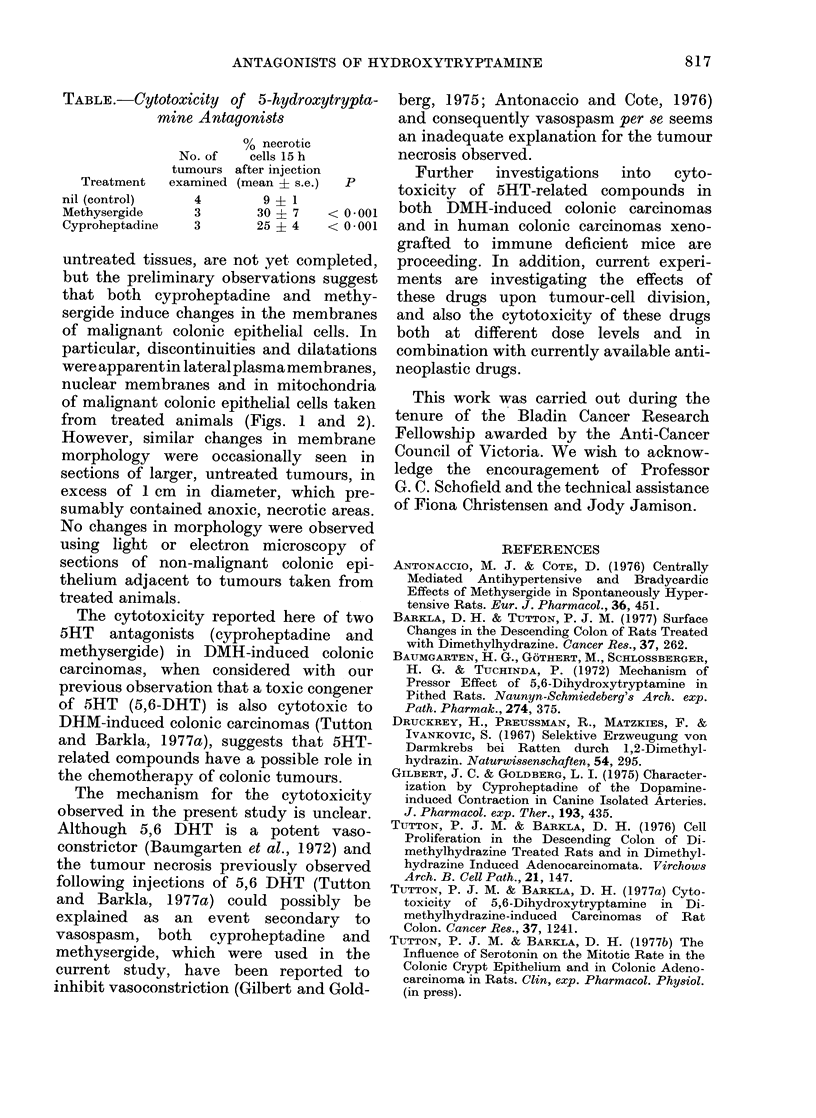# Cytotoxicity of cyproheptadine and methysergide to chemically induced carcinomas of rat colon.

**DOI:** 10.1038/bjc.1977.267

**Published:** 1977-12

**Authors:** D. H. Barkla, P. J. Tutton

## Abstract

**Images:**


*Br. J. Cancer* (1977) **36**, 814

***Short Communication***

### CYTOTOXICITY OF CYPROHEPTADINE AND METHYSERGIDE TO CHEMICALLY INDUCED CARCINOMAS OF RAT COLON

D. H. BARKLA AND P. J. M. TUTTON

*From the Department of Anatomy, Monash University, Clayton, Victoria* 3168, *Australia*

Received 14 June 1977 Accepted 14 July 1977

THE EXPERIMENTS described were prompted by two recent observations made in this laboratory. The first was that i.p. injections of small doses (10 $\mu$g/kg) of 5-hydroxytryptamine (5HT) increased the mitotic rate in 1,2-dimethylhydrazine (DMH)-induced carcinomas of rat colon, but were without effect on the mitotic rate in the adjacent non-malignant colonic epithelium (Tutton and Barkla, 1977*b*). The second observation was that i.p. injections of 5,6-dihydroxytryptamine, a toxic congener of 5HT (40 mg/kg) were cytotoxic to malignant cells in DMH-induced colonic carcinomas but non-toxic to the adjacent non-malignant colonic epithelium (Tutton and Barkla, 1977*a*). The present communication reports the cytotoxic effects of two 5HT antagonists, cyproheptadine and methysergide, on DHM-induced colonic carcinomas.

Colonic tumours were induced in male Sprague-Dawley rats, using 1,2-dimethylhydrazine (DMH) as described previously (Druckrey *et al.*, 1967; Tutton and Barkla, 1976). Tumour-bearing animals were given i.p. injections of either cyproheptadine (Merk Sharp & Dohme (Australia) Pty. Ltd, 1 mg/kg, 3 rats) or methysergide (Sandoz Australia Pty. Ltd, 0·1 mg/kg, 3 rats) and killed 15 h later by decapitation.

The doses used are similar to those reported previously as producing pharmacological blockade of peripheral 5HT receptors (Baumgarten *et al.*, 1972). Whilst the treatment duration for maximum effect has yet to be determined, 15 h was chosen as being a suitable time for identification of acutely necrotic cells *in situ* before possible phagocytosis or loss of necrotic cells into the intestinal lumen.

Specimens of descending colon and tumours of transverse or descending colon were prepared for light microscopic examination as described previously (Tutton and Barkla, 1976). Corresponding tissues from 4 tumour-bearing rats not treated with 5HT-antagonists were also prepared for light microscopy and served as controls. For electron microscopy, specimens of descending colon and tumours of transverse or descending colon were taken from experimental animals killed at 1 and 2 h after treatment with either cyproheptadine or methysergide, and prepared for electron microscopy as described previously (Barkla and Tutton, 1977). Corresponding tissues from untreated tumour-bearing rats were also prepared for electron microscopy using the same methods and served as controls. Histological sections of all tumours were examined initially at $\times$ 125 magnification, and those few tumours showing a histological organization other than well differentiated adenocarcinomas were discarded from the study.

Estimation of necrotic and non-necrotic cell numbers in histological sections of both control and experimental animals were made in the following way. Sections of tumours were examined at $\times$ 400 magnification and the numbers of necrotic and non-necrotic cells per visual field were counted using an eyepiece with a

FIG. 1.—Electron micrograph of the apical portions of several tumour cells abutting onto a glandular lumen (containing spiral micro-organisms and taken 1 h after treatment with cyproheptadine (1 mg/kg). Observe the discontinuous lateral plasma membranes and ribosomes in intercellular spaces (asterisks) and the dilated nuclear membrane (arrows). × 16,000.

rectangular graticule. Each successive visual field at an interval of 0·3 mm along mutually perpendicular axes extending from edge to edge of the histological section was examined in this way, and the mean percentage of necrotic cells per tumour was calculated. Between 1200 and 1800 cells were scored per tumour. Only cells with a distinctly pyknotic nucleus, that is, one lacking the normal vesicular

FIG. 2.—Electron micrograph of necrotic tumour cells taken 2 h after treatment with cyproheptadine (1 mg/kg). The lumen (L) of the tumour acinus can be seen in the upper right hand corner of the micrograph. Dilated mitochondria (arrows) and cytoplasmic vesicles are apparent. × 4800.

chromatin pattern, were recorded as necrotic. Results from each group of tumours treated in a particular way were pooled and the mean and standard error for the percentage of necrotic cells were calculated. The statistical significance of the apparent difference between treatment means was estimated using Students' $t$ test. The results are presented in the Table.

The observations, using electron microscopy of thin sections of treated and

TABLE.—*Cytotoxicity of 5-hydroxytryptamine Antagonists*

| Treatment | No. of tumours examined | % necrotic cells 15 h after injection (mean ± s.e.) | *P* |
|---|---|---|---|
| nil (control) | 4 | 9 ± 1 | |
| Methysergide | 3 | 30 ± 7 | < 0·001 |
| Cyproheptadine | 3 | 25 ± 4 | < 0·001 |

untreated tissues, are not yet completed, but the preliminary observations suggest that both cyproheptadine and methysergide induce changes in the membranes of malignant colonic epithelial cells. In particular, discontinuities and dilatations were apparent in lateral plasma membranes, nuclear membranes and in mitochondria of malignant colonic epithelial cells taken from treated animals (Figs. 1 and 2). However, similar changes in membrane morphology were occasionally seen in sections of larger, untreated tumours, in excess of 1 cm in diameter, which presumably contained anoxic, necrotic areas. No changes in morphology were observed using light or electron microscopy of sections of non-malignant colonic epithelium adjacent to tumours taken from treated animals.

The cytotoxicity reported here of two 5HT antagonists (cyproheptadine and methysergide) in DMH-induced colonic carcinomas, when considered with our previous observation that a toxic congener of 5HT (5,6-DHT) is also cytotoxic to DHM-induced colonic carcinomas (Tutton and Barkla, 1977*a*), suggests that 5HT-related compounds have a possible role in the chemotherapy of colonic tumours.

The mechanism for the cytotoxicity observed in the present study is unclear. Although 5,6 DHT is a potent vasoconstrictor (Baumgarten *et al.*, 1972) and the tumour necrosis previously observed following injections of 5,6 DHT (Tutton and Barkla, 1977*a*) could possibly be explained as an event secondary to vasospasm, both cyproheptadine and methysergide, which were used in the current study, have been reported to inhibit vasoconstriction (Gilbert and Goldberg, 1975; Antonaccio and Cote, 1976) and consequently vasospasm *per se* seems an inadequate explanation for the tumour necrosis observed.

Further investigations into cytotoxicity of 5HT-related compounds in both DMH-induced colonic carcinomas and in human colonic carcinomas xenografted to immune deficient mice are proceeding. In addition, current experiments are investigating the effects of these drugs upon tumour-cell division, and also the cytotoxicity of these drugs both at different dose levels and in combination with currently available antineoplastic drugs.

This work was carried out during the tenure of the Bladin Cancer Research Fellowship awarded by the Anti-Cancer Council of Victoria. We wish to acknowledge the encouragement of Professor G. C. Schofield and the technical assistance of Fiona Christensen and Jody Jamison.

### REFERENCES

ANTONACCIO, M. J. & COTE, D. (1976) Centrally Mediated Antihypertensive and Bradycardic Effects of Methysergide in Spontaneously Hypertensive Rats. *Eur. J. Pharmacol.*, **36**, 451.

BARKLA, D. H. & TUTTON, P. J. M. (1977) Surface Changes in the Descending Colon of Rats Treated with Dimethylhydrazine. *Cancer Res.*, **37**, 262.

BAUMGARTEN, H. G., GÖTHERT, M., SCHLOSSBERGER, H. G. & TUCHINDA, P. (1972) Mechanism of Pressor Effect of 5,6-Dihydroxytryptamine in Pithed Rats. *Naunyn-Schmiedeberg's Arch. exp. Path. Pharmak.*, **274**, 375.

DRUCKREY, H., PREUSSMAN, R., MATZKIES, F. & IVANKOVIC, S. (1967) Selektive Erzweugung von Darmkrebs bei Ratten durch 1,2-Dimethylhydrazin. *Naturwissenschaften*, **54**, 295.

GILBERT, J. C. & GOLDBERG, L. I. (1975) Characterization by Cyproheptadine of the Dopamine-induced Contraction in Canine Isolated Arteries. *J. Pharmacol. exp. Ther.*, **193**, 435.

TUTTON, P. J. M. & BARKLA, D. H. (1976) Cell Proliferation in the Descending Colon of Dimethylhydrazine Treated Rats and in Dimethylhydrazine Induced Adenocarcinomata. *Virchows Arch. B. Cell Path.*, **21**, 147.

TUTTON, P. J. M. & BARKLA, D. H. (1977*a*) Cytotoxicity of 5,6-Dihydroxytryptamine in Dimethylhydrazine-induced Carcinomas of Rat Colon. *Cancer Res.*, **37**, 1241.

TUTTON, P. J. M. & BARKLA, D. H. (1977*b*) The Influence of Serotonin on the Mitotic Rate in the Colonic Crypt Epithelium and in Colonic Adenocarcinoma in Rats. *Clin, exp. Pharmacol. Physiol.* (in press).